# A fast reliability assessment method using optimal basis for integrated community energy systems

**DOI:** 10.1371/journal.pone.0342059

**Published:** 2026-02-05

**Authors:** Jiangang Lu, Ruifeng Zhao, Wenxin Guo, Qian Li, Zeyu Liu, Kai Hou, Hao Wu, Yuli Liu

**Affiliations:** 1 Electric Power Dispatching & Control Center of Guangdong Power Grid Co., Ltd, Guangzhou, China; 2 State Key Laboratory of Intelligent Power Distribution Equipment and System, Tianjin University, Tianjin, China; Shanghai University of Electric Power, CHINA

## Abstract

Integrated Community Energy Systems (ICES) aim to optimize energy efficiency through the integration of diverse energy resources, encompassing electricity, heating systems, and natural gas. However, the rapid integration of renewable energy sources and the rising energy demands pose significant challenges in evaluating the reliability of ICES. This difficulty arises from the need to evaluate numerous system states to determine the minimal load curtailment. To address this issue, we propose a method based on optimal bases to enhance computational efficiency in the reliability assessments of ICES. The optimal load curtailment model is developed to facilitate system state evaluation, accounting for variations in load levels and renewable generation. Subsequently, the optimal basis is employed to accelerate this evaluation process. By matching most system states with their corresponding optimal basis based on the optimality criterion, efficient computation of optimal load curtailment is achieved through matrix multiplications, eliminating the need for time-consuming optimization algorithms. The efficacy of the optimal basis-based method is validated through comprehensive case studies.

## Introduction

Energy is crucial for maintaining societal stability and promoting economic development [1,2]. The integration of diverse energy networks offers significant advantages in addressing challenges such as energy shortages and environmental concerns, as exemplified by integrated community energy systems (ICES) [3,4]. These systems primarily rely on renewable energy sources and integrate combined heat and power (CHP) systems as the main units for energy supply. Effective coordination across these varied energy systems facilitates the maximized utilization of available energy resources [5]. The energy hub (EH) model, represented through a coupling matrix, quantitatively describes the multi-energy flow of electricity, gas, and heating [6]. It facilitates multi energy flow calculations, planning and operation optimizations [7], and supports demand response [8,9], energy storage [10,11], renewable energy [12], and electrical vehicles [13,14].

The ICES integrates multiple energy sources, offering flexible and efficient multi-energy supply solutions to meet varying energy demands [15]. However, this integration introduces new challenges related to system reliability [16,17]. To ensure the reliability of ICES, a comprehensive assessment that quantifies the reliability of both individual equipment and the overall system is required. The impact of CHP units on the reliability of ICES is typically evaluated using the continuous Markov method [18]. Similarly, this approach is also employed to assess the effect of combined cooling, heating, and power (CCHP) units on ICES reliability [19]. Assessments at the equipment-level have shown that integrating heat pumps and air conditioning systems into multi-energy systems can enhance their overall efficiency [20]. Ref. [21] introduces a Monte Carlo Simulation (MCS) approach, based on the EH model, which is designed for urban energy systems and supports higher penetration levels of renewable energy. Ref. [22] employs a sequential MCS approach to evaluate the reliability of integrated power-gas systems. Ref. [23] proposes a sequential MCS method that models component behaviour based on historical states, demonstrating the effect of the simultaneous presence of power-to-gas(P2G) and CHP technologies on reliability of ICES. While these simulation-based methods provide accurate reliability assessments, they require evaluating numerous system states leading to substantial computational burden. Furthermore, uncertainties, such as load level and renewable generation variations, are considered in ICES reliability assessment [24,25]. To address these uncertainties, several methods have been employed, such as k-means clustering, Wasserstein generative adversarial networks [26], stochastic optimization [27], and two-stage robust optimization [28], yet these approaches further increase the number of system states to evaluate. Therefore, developing computationally efficient reliability assessment methods that can handle large-scale state evaluations under uncertainties is critical for practical ICES applications.

Various methods have been suggested to improve the efficiency of ICES reliability assessments by reducing the number of system states that need to be evaluated. For example, Ref. [29] proposes a quasi-sequential MCS method integrated with a hierarchical decoupling optimization framework. Ref. [30] proposes a Markov model consisting of eight states to assess the reliability indices of multi-energy systems. The traditional Universal Generating Function (UGF) method is extended to integrated energy system by combining the contingencies of coupling devices and reducing the number of scenarios that require evaluations [31]. Moreover, a fuzzy UGF (FUGF) technique is introduced to evaluate the uncertainties of load and renewable generations in the reliability assessment [32]. However, these state reduction methods may sacrifice assessment completeness or accuracy by limiting the scenarios considered.

An alternative approach to improving the efficiency of optimal load curtailment (OLC) computations is through acceleration techniques. The multi-parametric linear programming method enables sensitivity analysis by pre-computing solution structures across parameter spaces, alleviating computational burden in iterative OLC solving [33]. Additionally, the impact increment method is introduced to adjust the influence of higher-order failure states, redirecting their effects toward those of lower-order states [34]. Integrating smart agent communication (SAC) techniques into the ICES reliability assessment framework is another promising method to enhance evaluation efficiency [35]. Moreover, data-driven methods have demonstrated remarkable acceleration potential. Graph neural networks achieve significant computational speedup compared to traditional solvers while maintaining high accuracy for large-scale systems [36], and active learning frameworks substantially reduce training data requirements through strategic sample selection [37]. Other data-driven approaches, including, Bayesian network [38] and, Hamming clustering algorithms [39], are also effective for accelerating computations. While these acceleration techniques show promise, data-driven methods require extensive training datasets and may face generalization challenges when system configurations change, whereas parametric programming methods are typically limited to specific problem structures. More importantly, most existing acceleration approaches either approximate the optimal solution or require problem reformulation, potentially sacrificing the accuracy essential for reliability assessment. As uncertainties in load levels and renewable generation increase alongside ICES structural complexity, there remains a critical need for acceleration methods that maintain optimization accuracy while dramatically reducing computational time.

This paper introduces a novel method for ICES reliability assessment that utilizes optimal basis (OB) techniques to efficiently compute optimal load curtailment. We propose an optimal load curtailment model to analyse how fluctuations in load and renewable generation impact system reliability. By employing optimal bases, the proposed method directly calculates optimal load curtailment, thus bypassing the need for time-intensive optimization processes while preserving solution precision. The main contributions of this work are summarized as follows:

1) A new optimal load curtailment model is developed to assess the effects of equipment failures, load variations, and renewable energy fluctuations on ICES reliability.
2) The use of optimal-basis-based functions replaces the numerous optimization steps typically involved in state evaluations, significantly accelerating the process of solving optimal load curtailment problems.
3) Optimality criteria are applied to link system states with their corresponding optimal bases, ensuring the precision and reliability of the solutions derived from the proposed method.

The structure of the paper is outlined as follows: The theoretical foundations of optimal bases are first presented. Following this, the proposed method for reliability assessment based on these optimal bases is outlined. Finally, a case study is included to illustrate the effectiveness of the proposed method in the reliability assessment of ICES.

### Optimal basis theory

The linear programming problem consists of an objective function, decision variables, and a set of constraints. This can be represented in standard form, with Equation (1) illustrating the linear programming model.


$$\begin{array}{c} min\;\;z=c^{T}x\\ s.t.\;\;Ax=b,\;\;x\geq 0 \end{array}$$ (1)

where ***A*** represents an *m* × *n* coefficient matrix of constraints; ***x*** is an *n*-vector of decision variable; ***b*** is an *m*-vector that includes the resource coefficients located on the right-hand side of the linear constraints; *z* represents the objective value, which is the value that the model is trying to minimize; ***c*** is an *n*-vector of coefficients for the objective function, indicating the significance of each decision variable in achieving the objective.

By using optimal bases, we can directly obtain the optimal value of the objective function,


$$z=c_{B}B^{\text{-1}}b$$ (2)

where ***B*** represents an optimal basis, which consists of *m* columns from the matrix ***A***; ***c***_*B*_ is the *m*-vectors of ***c*** corresponding to ***B***.

Evaluating the reliability of ICES involves analyzing numerous system states, which are influenced by variations in load and renewable generation. Consequently, repeatedly solving the linear programming problems for each state can be computationally demanding. The use of optimal bases provides an efficient way to speed up these computations, as it allows for the direct calculation of the optimal solution using the linear functions in Equation (2), based on the optimal bases. This significantly enhances the efficiency of the ICES reliability assessment.

## Optimal-basis-based reliability assessment method

### Framework of ICES reliability assessment

The ICES reliability assessment involves three main steps: system state generation, system state evaluation, and reliability indices calculation.

### System state generation

**Fig 1:**
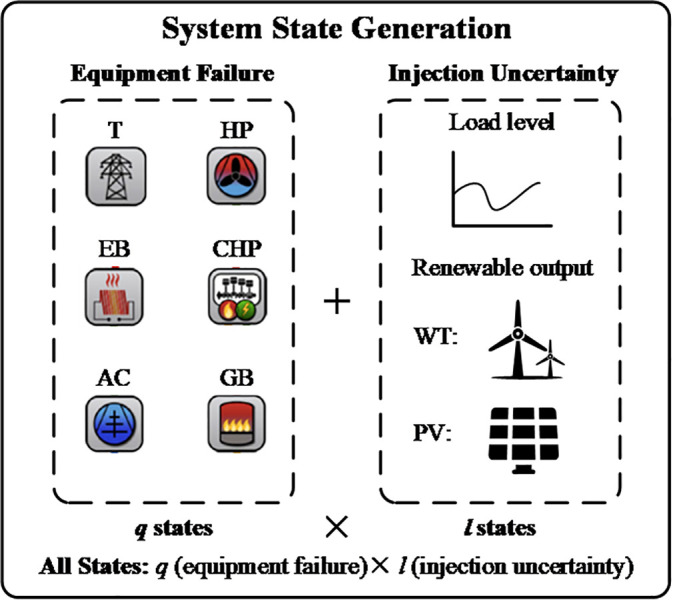
The process of system state generation.

Fig 1 shows the process of generating system states for the reliability assessment of ICES. These states are created by combining injection states and equipment failure states. Various equipment failures are considered in the ICES, including failures in the electricity transmission line (TLe), electric boilers (EB), heat pumps (HP), gas boilers (GB), air conditioners (AC), and CHP systems. Injection uncertainties, such as changes in load levels and renewable generation outputs, also contribute to the system state variations.

### System state evaluation

**Fig 2:**
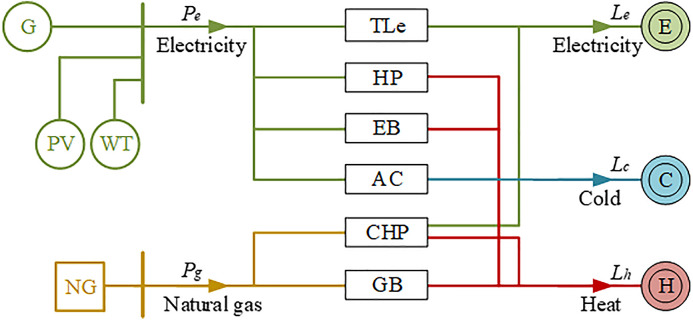
The structure of ICES.

To evaluate these system states, the OLC model must be applied to all the system states shown in Fig 2 This model can adjust energy purchases from the networks, reschedule equipment outputs, and calculate the necessary energy curtailments for the ICES.

Unlike traditional power systems, the ICES needs to coordinate different energy equipment to supply multiple types of loads. Fig 2 illustrates the structure of an ICES. Heat pumps (HP) and electric boilers (EB) transform electricity into heat, whereas gas boilers (GB) rely on gas as a fuel source for heat generation. Air conditioners (AC) convert electricity into cooling. Additionally, the CHP system generates both electricity and heat concurrently. The energy supply is derived from multiple sources: natural gas is obtained from the gas station (NG), while electricity is supplied by the grid (G), photovoltaics (PV), and wind turbines (WT). The Renewable energy penetration reflects the extent of renewable energy integration, which is defined as the share of total generation capacity provided by wind and solar power, as shown in Equation (3),


$$p_{re}=\frac{P_{PV}+P_{WT}}{P_{G}+P_{PV}+P_{WT}}$$ (3)

where *p*_*re*_ denotes the level of renewable energy penetration; *P*_*G*_ is the maximum capacity of electricity supplied by the grid; *P*_*PV*_ and *P*_*WT*_ are the capacities of PV and WT.

The goal of the optimal load curtailment model is to minimize the overall reduction in electricity, heat, and cooling loads within the ICES, as described in Equation (4). The objective function includes weighted factors for electricity, heat, and cooling,


$$min\;\;wS=w_{e}S_{e}+w_{h}S_{h}+w_{c}S_{c}$$ (4)

where ***S*** = [*S*_*e*_, *S*_*h*_, *S*_*c*_]^T^ is the load curtailment of electricity, heat, and cooling; ***w*** = [*w*_*e*_, *w*_*h*_, *w*_*c*_] is the weight vector of electricity, heat, and cooling.

The multi-energy balance constraint in the ICES is represented as shown in Equation (5),


L−CP=S (5)

where ***L*** = [*L*_*e*_, *L*_*h*_, *L*_*c*_]^T^ is the load of electricity, heat, and cooling energy; ***P***= [*P*_*e_T*_, *P*_*e_HP*_, *P*_*e_EB*_, *P*_*e_AC*_, *P*_*g_CHP*_, *P*_*g_GB*_]^T^ represents the input power from various equipment such as the T, HP, EB, AC, CHP, and GB; ***C*** serves to quantify the mathematical relationships that couple various types of energy, as shown in Equation (6),


$$C\text{=}\left[\begin{array}{cccccc} \eta_{T} & 0 & 0 & 0 & \eta_{CHPe} & 0\\ 0 & \eta_{HP} & \eta_{EB} & 0 & \eta_{CHPh} & \eta_{GB}\\ 0 & 0 & 0 & \eta_{AC} & 0 & 0 \end{array}\right]$$ (6)

where *η*_*T*_ is the transmission efficiency of electricity; *η*_*HP*_, *η*_*EB*_, *η*_*GB*_, and *η*_*AC*_ are the conversion efficiencies of HP, EB, GB, and AC; *η*_*CHPe*_ and *η*_*CHPh*_ represent the electricity and heat conversion efficiencies of the CHP, where *η*_*CHPe*_/ *η*_*CHPh*_ is fixed.

The load shedding constraint as shown in Equation (7),


0≤S≤L,\hspace{0.5em}i∈N (7)

The equipment capacity, energy transmission efficiency, and conversion efficiency impose additional constraints, as shown in Equation (8),


$$\eta P\leq P_{cap}$$ (8)

where ***P***_*cap*_= [*P*_*cap_T*_, *P*_*cap_HP*_, *P*_*cap_EB*_, *P*_*cap_AC*_, *P*_*cap_CHP*_, *P*_*cap_GB*_]^T^ represents the equipment capacity, with the CHP capacity being defined by its maximum heat output; ***η*** is the efficiency matrix of equipment, as shown in Equation (9),


$$\eta =\left[\begin{array}{cccccc} \eta_{T} & \eta_{HP} & \eta_{EB} & \eta_{AC} & \eta_{CHPh} & \eta_{GB} \end{array}\right]\;\;I$$ (9)

where ***I*** is the identity matrix.

The energy input constraint is described as shown in Equation (10),


$$TP\leq P_{EG}$$ (10)

where ***P***_*EG*_= [*P*_*G*_ + *P*_*PV*_ + *P*_*WT*_, *P*_*NG*_]^T^; *P*_*EG*_ represents the combined capacity of the grid, PV, WT, and natural gas sources; ***T*** is the matrix that characterizes energy flows between components in the ICES, as shown in Equation (11).


$$T=\left[\begin{array}{cccccc} 1 & 1 & 1 & 1 & 0 & 0\\ 0 & 0 & 0 & 0 & 1 & 1 \end{array}\right]$$ (11)

After adding slack variables ***y***, the OLC model can be reformulated into a standard linear programming model. The parameters are follows in Equations (12)-(15),


$$x=[P,\;\;S,\;\;y{]}^{\text{T}}$$ (12)

$$c=[0,\;\;w,\;\;0{]}^{\text{T}}$$ (13)

$$b=[L,\;\;L,\;\;P_{cap},\;\;P_{EG}{]}^{\text{T}}$$ (14)

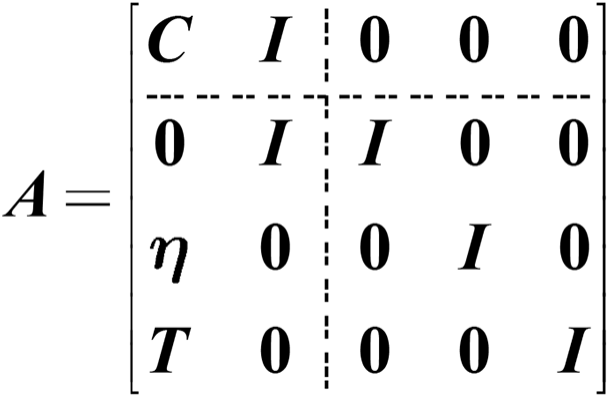
 (15)

### Reliability indices calculation

The reliability of ICES is evaluated based on the Expected Energy Not Supplied (EENS), which accounts for the total energy that is curtailed in the system, as expressed in Equations (16) and (17). The EENS is calculated by considering the optimal load curtailment *I*(*s*) for each system state *s* and the probability of occurrence of each state *P*(*s*). The reliability assessment is conducted over a defined time scale *T*, with the EENS calculated separately for electricity, heat, and cooling energy.


$$\text{EENS}=T\sum_{s\in \Omega }I\left(s\right)P\left(s\right)$$ (16)

$$P\left(s\right)=\prod_{i\in s}a_{i}\prod_{j\notin s}u_{j}$$ (17)

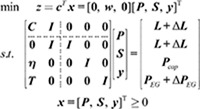
 (18)

where Ω represents the set of all possible states that have been considered; *a*_*i*_ and *u*_*i*_ are the probabilities of equipment *i* being available and unavailable, respectively, with *a*_*i*_ + *u*_*i*_ = 1.

### Optimal-basis-based OLC computation method

In traditional evaluation methods, analyzing each system state requires repeatedly solving the optimal load curtailment (OLC) problems, which can be very time-consuming because of the large number of states that need to be analyzed. As shown in Fig 1, the main cause of variation among system states comes from injection uncertainties. Moreover, the load curtailment due to these small differences can be directly determined using optimal bases. Therefore, an approach based on optimal bases is proposed to speed up the OLC calculations for injection uncertainties by avoiding the need to solve the OLC models repeatedly for each system state.

Injection uncertainties arise from variations in load levels and renewable energy generation, which influence the values of ***L*** and ***P***_*EG*_ in (14). These variations only affect the vector ***b*** in the equations (12) – (15). As a result, the ICES optimal load curtailment model with injection uncertainties can be expressed as Equation (18),

Then, the functions based on optimal bases to calculate the optimal load curtailment are, as shown in Equation (19),


$$z=c_{B}B^{\text{-1}}(b+\Delta b)$$ (19)

where Δ***b*** includes the changes in load levels Δ***L*** and renewable energy generations Δ***P***_*EG*_; ***B*** is the optimal basis.

Moreover, the optimality criteria (20) and (21) are used to test the correctness of the optimal bases for the new linear programming (LP) problem in (18),


$$x_{B}=B^{\text{-1}}(b+\Delta b)\geq 0$$ (20)

$$\sigma =c-c_{B}B^{\text{-1}}A\geq 0$$ (21)

where the optimal basis ***B*** for the initial OLC problem remains the same in the new OLC problem since the matrix ***A*** remains constant.

If the optimality criteria (20) and (21) are satisfied, ***B*** becomes the optimal basis of the new LP problem (18) and can be used to compute the OLC using (19), aligning it with the new system state. If the optimality conditions are not satisfied, the new system state’s OLC must be solved using linear programming optimization. The resulting optimal basis is then added to the optimal basis set (OB-Set) for future use.

Algorithm 1: System State Evaluation

1:   **Inputs:** OLC problem of a state ***s***, OB-Set, flag = 0

2:   *N*_*s*_ (the number of OB in OB-Set)

3:   *N*_*u*_ (the number of times that OB has been matched)

4:   *N*_*m_max*_ (the maximum matching times of state ***s***)

5:   flag = 0 (*Initialized to 0, indicating that the solution has not yet been found.*)

6:   *i* ← *N*_*s*_

7:   **while** *i ≥* 1 and (*N – i ≤ N*_*m_max*_) **do**

8:   **if** optimality criteria (20) and (21) are true **then**

9:   flag = 1

10: OB of state ***s*** ∈ OB-set

11: *N*_*u*_*(i)* = *N*_*u*_*(i) +* 1

12: Sort OB-set in ascending order of *N*_*OB*_

13: OLC is calculated by (19)

14: **break**

15: **end if**

16: **end while**

17: *i* = *i* + 1

18: **if** flag is false **then**

19: *N*_*s*_ = *N*_*s*_ + 1

20: OLC is calculated by LP optimizations

21: B is saved in the OB-set (*N*_*s*_)

22: **end**

23: **Outputs:** Optimal load curtailment of state ***s***, updated OB-sets, *N*_*u*_, and *N*_*s*_

Moreover, an ordering approach for the OB-Set is used to accelerate the matching process, which involves sorting the optimal bases based on the number of times they have been matched. The higher-ranked optimal bases will be first checked for the optimality criteria. Additionally, the maximum matching times *N*_*m_*max_ are set to prevent excessive matches. The system state evaluation process is formulated in Algorithm 1.

In most cases, the same optimal basis can be used for system states with the same equipment failure. This enables the OB-based function in equation (19) to compute the optimal load curtailment, offering a more cost-effective alternative to the expensive LP optimization method. This method can significantly reduce the time needed to solve the OLC model, improving the efficiency of the ICES reliability assessment.

### Overview of the proposed methodology

**Fig 3:**
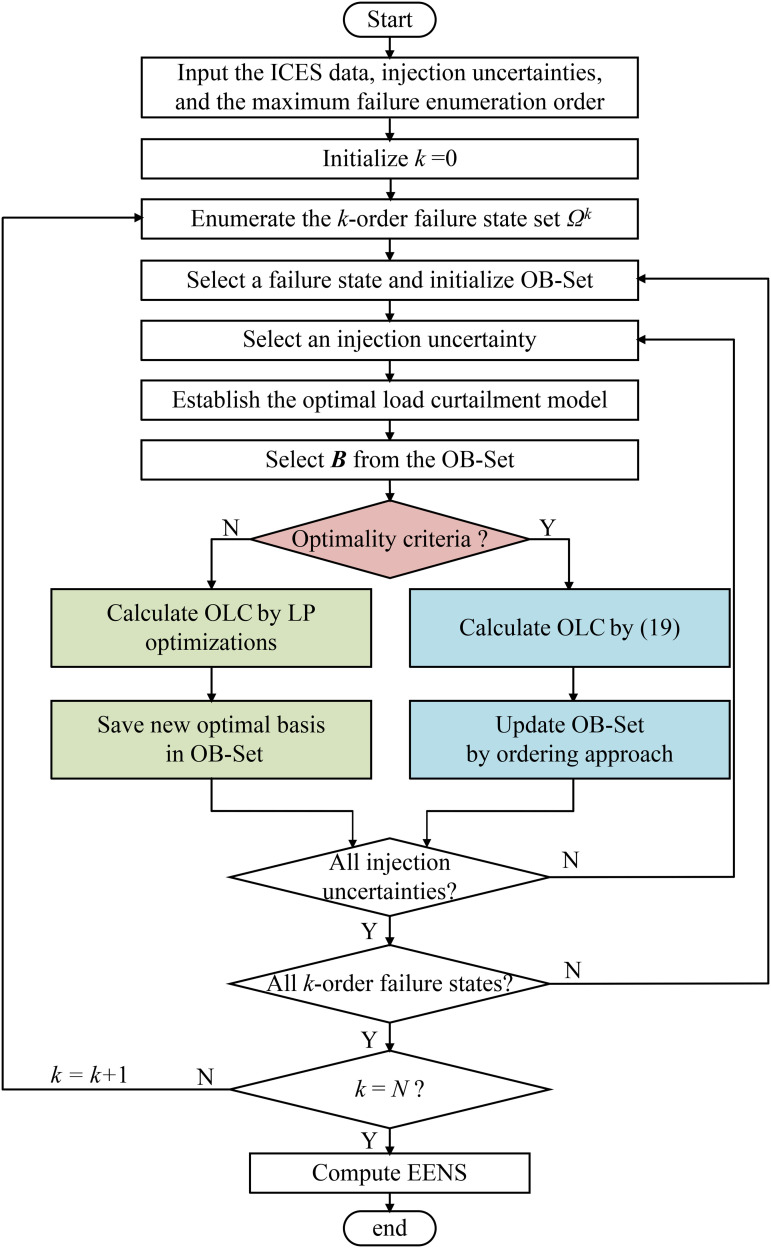
The overall process of OB.

This study introduces a fast reliability assessment method based on optimal bases for integrated community energy systems. The general procedure of the proposed OB method is shown in Fig 3, accompanied by the following step-by-step process.

**Step 1**: Input the ICES data and injection uncertainties. Set the maximum failure enumeration order *N*.

**Step 2**: Initiate the failure order *k*, by setting it to 0

**Step 3**: Enumerate the *k*-order failure state set *Ω*^*k*^

**Step 4**: Select an equipment failure state from the set *Ω*^*k*^ and initialize the optimal bases set (OB-Set).

**Step 5**: Select an injection uncertainty and find a new state

**Step 6**: Establish the OLC model for the new state

**Step 7**: Select the optimal basis matrix ***B*** from the OB-Set.

**Step 8**: Validate whether the optimality criteria (20) and (21) are satisfied. If yes, obtain the OLC of the new state using the selected optimal bases and update the OB-Set by sorting it. Otherwise, calculate the OLC of the new state using LP optimization and store its ***B*** into the OB-Set.

**Step 9**: Determine whether all injection uncertainties have been considered. If they are, proceed to the next step. If not, return to Step 5.

**Step 10**: Determine whether all *k*-order failure states have been assessed. If they are, proceed to the next step. If not, return to Step 4.

**Step 11**: Determine whether *k* is equal to *N*. If so, proceed to the next step. If not, *k = k* + 1 and return to Step 3.

**Step 12**: Compute the reliability index EENS

### Case study

The evaluation of the proposed method involves testing it on an ICES system, which represents an electric-heating-cooling system. This system is shown in Fig 2, and the specific parameters are listed in Table A.1 of the S1 Appendices [29]. The maximum electricity capacity from the grid is 1.0 MW, while that of the natural gas station is 0.6 MW. The peak loads of electricity, heat, and cooling are 0.72, 1.06, and 0.53 MW, respectively. The load curves are taken from National Renewable Energy Laboratory [33]. The weight factors of electricity, heat, and cooling are ***w*** = [*w*_*e*_, *w*_*h*_, *w*_*c*_] = [0.4, 0.3, 0.3].

The reliability assessment of the ICES is conducted using the state enumeration (SE), MCS, and the proposed OB methods. The MCS uses a sample size of 5 × 10^7^, and its results serve as the standard for verifying the accuracy of the reliability results. The efficiency of SE is compared with the OB method. The maximum failure enumeration order *N* is set to 3.

The effectiveness of the proposed OB method is evaluated through two distinct scenarios. In Scenario 1 (S1), the ICES operates without any renewable energy generation, while in Scenario 2 (S2), PV and WT are integrated into the system.

### Accuracy and efficiency

As demonstrated by the results of S1 in Table 1, the proposed OB method dramatically improves the efficiency of ICES reliability evaluation. Specifically, the OB method is approximately 54 times faster than the SE method, while maintaining the reliability indices with a relative error of under 5%. It is also noted that the reliability indices for heat (EENS_h_) and cooling (EENS_c_) are both higher than those for electricity (EENS_e_). This suggests that electricity loads demonstrate better reliability performance, owing to the higher heat load demand (1.06 MW) compared to the electricity demand (0.72 MW). Meanwhile, the higher reliability indices for cooling load are due to the structure of ICES, where only the air conditioner (AC) provides cooling energy.

**Table 1:** Reliability assessment of ICES.

Scenario	Method	EENS (MWh/y)			CPU Time (s)
		EENS_e_	EENS_h_	EENS_c_	
S1	MCS	0.329	0.506	0.495	6275.0
	SE	0.310	0.491	0.483	75.7
	OB	0.310	0.491	0.483	1.4
S2	MCS	0.622	0.491	0.486	6621.2
	SE	0.627	0.492	0.483	78.7
	OB	0.627	0.492	0.483	1.3

The results of S2 in Table 1 further confirm the effectiveness of the proposed OB method. In this scenario, the OB method is 60 times faster than the SE method, with a relative error of less than 3%. However, the addition of renewable energy in S2 leads to a decrease in the overall reliability of the ICES. Specifically, the electricity reliability indices in S2 are nearly double those in S1.

Fig A.1 in the S1 Appendices shows that the OB and SE points are both within the MCS envelope, with the LM point being the leftmost. This highlights the significant speed and accuracy improvements the OB method provides compared to traditional approaches for assessing ICES reliability.

Table 2 provides the number of optimal bases for each failure order. The total number of system states is 8760 × (${\text{C}}_{\text{6}}^{\text{0}}+{\text{C}}_{\text{6}}^{\text{1}}+{\text{C}}_{\text{6}}^{\text{2}}+{\text{C}}_{\text{6}}^{\text{3}}$)= 367920. However, the OLC optimizations assess only 445 of these states, as indicated by the total number of optimal bases. On average, only 11 injection states fail to match the optimal bases during each failure state, meaning the optimal bases are effective in evaluating over 98% of the system states. As a result, the OB method is a more efficient alternative to the time-consuming task of solving the OLC model for most system states.

**Table 2:** Optimal basis number in S1.

Failure Order	Failure States Number	Total OB Number	Average OB number
0	1	9	9
1	6	78	13
2	15	182	12
3	20	176	9
All	41	445	11

### Impacts of renewable generations

Table 3 presents the impact of renewable energy on ICES reliability. As the penetration level rises, the reliability of electricity steadily decreases, whereas the reliability indices for heat and cooling remain unchanged. This occurs because renewable energy penetration primarily influences the electricity generation capacity. Given the ample supply of gas and the high efficiency of air conditioning, reducing electricity loads becomes a top priority. Fig A.2 in the S1 Appendices visually demonstrates the variation in reliability indicators, including EENS_e_, EENS_h_ and EENS_c_, under different levels of renewable energy penetration.

**Table 3:** The impact of renewable energy.

Penetration	Method	EENS (MWh/y)			CPU Time (s)
		EENS_e_	EENS_h_	EENS_c_	
0	MCS	0.329	0.506	0.495	6275.0
	SE	0.310	0.491	0.483	75.7
	OB	0.310	0.491	0.483	1.4
0.05	MCS	0.310	0.487	0.478	6523.4
	SE	0.310	0.491	0.483	76.8
	OB	0.310	0.491	0.483	1.3
0.10	MCS	0.320	0.491	0.476	6571.7
	SE	0.310	0.491	0.483	77.4
	OB	0.310	0.491	0.483	1.3
0.15	MCS	0.622	0.491	0.486	6621.2
	SE	0.627	0.492	0.483	78.7
	OB	0.627	0.492	0.483	1.3
0.20	MCS	3.276	0.485	0.477	6634.8
	SE	3.279	0.494	0.483	79.86
	OB	3.279	0.494	0.483	1.5
0.25	MCS	11.548	0.499	0.488	6658.7
	SE	11.575	0.498	0.483	80.50
	OB	11.575	0.498	0.483	1.7

Furthermore, for renewable energy penetration levels below 0.1, the reliability indices show only slight variations, suggesting that the system can effectively accommodate the utilization of these renewable energy sources. However, once the penetration exceeds 0.15, the electricity reliability index drops significantly. Therefore, the maximum renewable energy that the ICES can handle is about 15% of the total generation capacity.

### Impacts of energy conversion equipment

The energy conversion equipment is essential for the reliable operation of ICES. To assess the influence of this equipment on ICES reliability, multiple scenarios are presented in Table 4. The outcomes highlight the pivotal role of energy conversion devices in shaping the overall reliability of ICES.

**Table 4:** Reliability assessment results of different equipment.

Case	Energy Conversion Equipment	EENS (MWh/y)		
		EENS_e_	EENS_h_	EENS_c_
A0	Original Structure	0.310	0.491	0.483
A1	No HP units	0.310	56.092	0.483
A2	No Eb units	0.310	44.806	0.483
A3	No CHP units	0.401	23.006	0.483
A4	No GB units	0.310	48.362	0.483

The heat reliability indices of Case A1 - A4 are higher than the original Case A0, confirming that energy conversion equipment improves heat reliability. Among these cases, A1, A2, and A4 show higher heat indices than Case A3, suggesting that heat pumps (HP), electric boilers (EB), and gas boilers (GB) have a greater impact on ICES reliability. Since the heat generation in combined heat and power (CHP) systems is constrained by electricity production, the improvement in heat reliability is less significant in Case A3.

Furthermore, it is noteworthy that the electricity indices remain nearly unchanged across all four cases because most of the electricity load is met by the power grid. Additionally, since there are no alterations in AC, the cooling reliability indices also remain unaffected in all four cases.

These findings suggest that energy conversion equipment has the potential to enhance the reliability level by facilitating multi-energy integration and minimizing energy losses during the conversion process.

### Reliability enhancement strategies

A variety of strategies are utilized to enhance the reliability of integrated community energy systems. This study primarily investigates how equipment capacity affects the improvement of reliability in ICES. Table 5 illustrates the impact of different equipment capacities on the reliability of ICES, providing important insights into how equipment capacity influences system reliability.

**Table 5:** Reliability assessment results of capacity cases.

Case	Capacity	EENS (MWh/y)		
EENS_e_	EENS_h_	EENS_ca_
B0	Origin	0.310	0.491	0.483
B1	1.1 HP	0.310	0.491	0.483
	1.3 HP	0.310	0.415	0.483
	1.5 HP	0.310	0.415	0.483
B2	1.1 EB	0.310	0.491	0.483
	1.3 EB	0.310	0.384	0.483
	1.5 EB	0.310	0.320	0.483
B3	1.1 CHP	0.304	0.467	0.483
	1.3 CHP	0.294	0.420	0.483
	1.5 CHP	0.284	0.375	0.483
B4	1.1 GB	0.310	0.455	0.483
	1.3 GB	0.310	0.386	0.483
	1.5 GB	0.310	0.325	0.483

As shown in Table 5, there are four Cases B1 - B4, where the capacities of HP, EB, CHP, and GB are improved respectively. The results show that heat reliability decreases slightly with increasing equipment capacity, while electricity and cooling reliability remain stable due to sufficient electricity from the grid and fixed cooling outputs from AC.

Fig A.3 in the S1 Appendices shows that Cases B2 and B4 outperform Cases B1 and B3 in terms of reliability enhancement. It can be attributed to the higher heat efficiency of EB and GB in contrast to HP and CHP. The expansion of different equipment capacities has varying effects on reliability enhancement. Consequently, proper configuration and planning of equipment capacity emerge as crucial strategies for improving the reliability of ICES.

Increasing the capacity of CHP can improve the reliability of electricity in ICES with a high level of renewable energy sources. However, Case B3 shows that the expansion of CHP has little effect on electricity indices because its heating generation limits the electricity generation of CHP. To address this, the heating generation of CHP can be reduced to allow more electricity generation. As shown in Table A.2 in the S1 Appendices , this remarkably improves the electricity reliability, thereby enhancing the ability of ICES to consume renewable energy.

The results of Tables 5 and A.2 in S1 Appendices demonstrate that the equipment expansion does not contribute to an enhancement in cooling reliability. To address this issue, more suitable maintenance plans can be implemented to shorten the AC repair time. The enhancement results are shown in Table A.3 in the S1 Appendices.

## Conclusion

This study presents a fast reliability assessment method using optimal bases for integrated community energy systems. The method entails developing an optimal load curtailment model for ICES to assess the effects of equipment failures and injection uncertainties on system reliability. The implementation of optimal bases allows for the fast calculation of the optimal load curtailment, eliminating the need for time-consuming optimization techniques.

A case study is performed to illustrate the enhanced efficiency of the proposed OB approach compared to the conventional SE method. The results demonstrate that the OB method offers a speed improvement of over 50 times compared to the SE method. Furthermore, optimal bases can be effectively matched with over 98% of the system states, significantly expediting computations using the optimal-bases-based functions. Additionally, the OB method is applied to investigate the effects of renewable energy generation and energy conversion equipment on ICES reliability. Several strategies are developed to enhance the reliability of electricity, heat, and cooling for ICES. Looking forward, the methodology can be extended to incorporate additional flexible resources, such as battery energy storage systems and demand-side management (DSM). These components are expected to further improve system flexibility, mitigate renewable energy intermittency, and enhance overall reliability and resilience. Overall, the OB method provides an efficient way to accelerate the reliability evaluation of ICES and formulate strategies for enhancing the overall system reliability and resilience.

### Nomenclature

**Table:** 

Acronyms	
ICES:	Integrated community energy systems
CHP:	Combined heat and power
EH:	Energy hub
CCHP:	Combined cooling, heating, and power
MCS:	Monte carlo simulation
UGF:	Universal generating function
FUGF:	Fuzzy UGF
OLC:	Optimal load curtailment
SAC:	Smart agent communication
OB:	Optimal basis
TLe:	Electricity transmission line
EB:	Electric boilers
HP:	Heat pumps
GB:	Gas boilers
AC:	Air conditioners
NG:	Gas station
G:	Grid
PV:	Photovoltaics
WT:	Wind turbines
EENS:	Expected energy not supplied
SE:	State enumeration
LP:	Linear programming
S1	Scenario 1
S2	Scenario 2
Variables	
***A***	The *m* × *n* coefficient matrix of constraints in the linear programming model
*a*_*i*_	The probability of equipment *I* being available
***B***	An optimal basis, which consists of *m* columns from the matrix *A*
***b***	The resource vector on the right-hand side of the constraints
***C***	The matrix representing the coupling of different energy forms
***c***	An *n*-vector of coefficients for the objective function
***c***_***B***_	The *m*-vector of *c* corresponding to the optimal basis *B*
***k***	The failure order
***L***	The load vector of electricity, heat, and cooling energy
***N***	The maximum failure enumeration order
P	The input power vector from various equipment
***P***_*cap*_	The equipment capacity vector
***P***_*EG*_	The combined capacity of the grid, PV, WT, and natural gas sources
*P*(*s*)	The probability of occurrence of system state *s*
*p*_*re*_	The level of renewable energy penetration
***S***	The load curtailment vector of electricity, heat, and cooling
***s***	A system state
***T***	The matrix that characterizes energy flows between components in the ICES
*u*_*i*_	The probability of equipment *I* being unavailable
***w***	The weight vector of electricity, heat, and cooling
***x***	An *n*-vector of decision variables
***y***	The slack variables
*z*	The objective value of the optimization model
***η***	The efficiency matrix of equipment
Δ***b***	A vector that includes the changes in load levels and renewable energy generation
σ	The parameter used to test the optimality criterion for the optimal basis
Ω	The set of all possible system states that have been considered

## Supporting information

S1 Appendices Table A.1 Equipment parameters. Table A.2 Reliability Assessment Results of CHP Expansion. Table A.3 Reliability enhancement through AC maintenance. Figure A.1. The comparison of three methods (S1). Figure A.2. EENS of the different renewable energy penetrations. Figure A.3. EENS indices of heat in four cases.
(DOCX)
