# Supplementary material for: A fast reliability assessment method using optimal basis for integrated community energy systems
Source: PLoS One. 2026 Feb 5;21(2):e0342059. doi: 10.1371/journal.pone.0342059 (PMC12875581; doi:10.1371/journal.pone.0342059)
Supplement: S1 Appendices — Table A.2 Reliability Assessment Results of CHP Expansion. Table A.3 Reliability enhancement through AC maintenance. Figure A.1. The comparison of three methods (S1). Figure A.2. EENS of the different renewable energy penetrations. Figure A.3. EENS indices of heat in four cases. (DOCX) [file pone.0342059.s001.docx]

**Appendices**

**Table A.1** Equipment parameters

| Equipment | Capacity (MW) | Efficiency | Failure rate(occ./year) | Repair time (h) |
| --- | --- | --- | --- | --- |
| TLe | 0.80 | 0.95 | 0.065 | 5 |
| HP | 0.20 | 3.00 | 0.065 | 100 |
| EB | 0.20 | 0.90 | 0.020 | 200 |
| AC | 0.30 | 2.00 | 0.030 | 200 |
| CHP | 0.40 | 0.40/0.30 | 0.030 | 200 |
| GB | 0.20 | 0.95 | 0.025 | 300 |

**Table A.2** Reliability Assessment Results of CHP Expansion

| Penetration | Capacity | EENS (MWh/y) | | |
| --- | --- | --- | --- | --- |
|  |  | EENS_e_ | EENS_h_ | EENS_c_ |
| 0.15 | Origin | 0.627 | 0.492 | 0.483 |
|  | 1.1 CHP | 0.230 | 0.488 | 0.483 |
|  | 1.3 CHP | 0.206 | 0.481 | 0.483 |
|  | 1.5 CHP | 0.185 | 0.473 | 0.483 |
| 0.20 | Origin | 3.279 | 0.494 | 0.483 |
|  | 1.1 CHP | 0.232 | 0.491 | 0.483 |
|  | 1.3 CHP | 0.209 | 0.483 | 0.483 |
|  | 1.5 CHP | 0.188 | 0.476 | 0.483 |
| 0.25 | Origin | 11.575 | 0.498 | 0.483 |
|  | 1.1 CHP | 0.240 | 0.494 | 0.483 |
|  | 1.3 CHP | 0.217 | 0.486 | 0.483 |
|  | 1.5 CHP | 0.195 | 0.479 | 0.483 |

**Table A.3** Reliability enhancement through AC maintenance

| Repair time (h) | EENS (MWh/y) | | |
| --- | --- | --- | --- |
|  | EENS_e_ | EENS_h_ | EENS_c_ |
| 200 | 0.310 | 0.491 | 0.483 |
| 180 | 0.310 | 0.491 | 0.435 |
| 160 | 0.310 | 0.491 | 0.387 |
| 140 | 0.310 | 0.491 | 0.338 |
| 120 | 0.310 | 0.491 | 0.290 |
| 100 | 0.310 | 0.491 | 0.242 |

**(a)** The relative error of EENS_e_

**(b)** The relative error of EENS_h_

**(c)** The relative error of EENS_c_

**Figure A.1.** The comparison of three methods (S1)

**Figure A.2.** EENS of the different renewable energy penetrations

**Figure A.3.** EENS indices of heat in four cases
